# Angiogenically active vascular endothelial growth factor is over-expressed in malignant human and rat prostate carcinoma cells

**DOI:** 10.1054/bjoc.2000.1122

**Published:** 2000-04-27

**Authors:** H J Chen, A T Treweeke, Y Q Ke, D C West, C H Toh

**Affiliations:** Departments of Haematology Pathology and Immunology, University of Liverpool, Duncan Building, Liverpool, L69 3BX, UK

**Keywords:** vascular endothelial growth factor, prostatic cell lines, over-expression, metastasis, isoforms

## Abstract

Vascular endothelial growth factor (VEGF) is one of the most potent factors for stimulating angiogenesis, an essential process required for expansion of primary tumour and dissemination of malignant cells. To investigate the possible role of VEGF in facilitating metastasis of prostate cancer via stimulating angiogenesis, we have used Northern and slot blotting, reverse transcription polymerase chain reaction, nucleotide sequence analysis and enzyme-linked immunosorbent assay to compare the VEGF expression in series of human and rat cell lines with either benign or malignant characteristics. We have also employed the chick chorioallantoic membrane (CAM) assay to measure the angiogenic activity of the VEGF derived from both benign and malignant cells. The level of VEGF mRNA expressed in the seven malignant human and rat cell lines is 3.5- to 10-fold higher than that expressed in the benign cell lines. The three metastatic variants, generated by transfection of a benign cell line with DNA extracted from prostate carcinoma cells, expressed 2.5 to 5 times more VEGF mRNA than their parental benign cells. While VEGF 121 and 165 were predominantly expressed by both the benign and malignant cells, the transcript representing VEGF 189 isoform was only detected in the malignant cells. At protein level, three human malignant cell lines produced more VEGF (2.7–7.9 ng ml^−1^) than the benign cell line (1.3 ng ml^−1^). CAM assay detected a VEGF-dependent angiogenic activity in the medium from malignant cells, but only a relatively weak VEGF-independent activity in the medium from benign cells. These results demonstrated that malignant cells did over-express VEGF and only the VEGF derived from malignant cells was angiogenically active. Thus, we suggest that the VEGF produced by malignant cells might play an important role in facilitating metastasis of prostatic cancer. © 2000 Cancer Research Campaign


*British Journal of Cancer* (2000) **82**(10), 1694–1701
© 2000 Cancer Research Campaign
DOI: 10.1054/ bjoc.2000.1122, available online at http://www.idealibrary.com on IDEAL®

### Angiogenically active vascular endothelial growth factor is over-expressed in malignant human and rat prostate carcinoma cells

**HJ Chen[1], AT Treweeke[1], YQ Ke[2], DC West[3] and CH Toh[1]**

Departments of [1]Haematology, [2]Pathology and [3]Immunology, University of Liverpool, Duncan Building, Liverpool L69 3BX, UK

**Summary** Vascular endothelial growth factor (VEGF) is one of the most potent factors for stimulating angiogenesis, an essential process required for expansion of primary tumour and dissemination of malignant cells. To investigate the possible role of VEGF in facilitating metastasis of prostate cancer via stimulating angiogenesis, we have used Northern and slot blotting, reverse transcription polymerase chain reaction, nucleotide sequence analysis and enzyme-linked immunosorbent assay to compare the VEGF expression in series of human and rat cell lines with either benign or malignant characteristics. We have also employed the chick chorioallantoic membrane (CAM) assay to measure the angiogenic activity of the VEGF derived from both benign and malignant cells. The level of VEGF mRNA expressed in the seven malignant human and rat cell lines is 3.5- to 10-fold higher than that expressed in the benign cell lines. The three metastatic variants, generated by transfection of a benign cell line with DNA extracted from prostate carcinoma cells, expressed 2.5 to 5 times more VEGF mRNA than their parental benign cells. While VEGF 121 and 165 were predominantly expressed by both the benign and malignant cells, the transcript representing VEGF 189 isoform was only detected in the malignant cells. At protein level, three human malignant cell lines produced more VEGF (2.7–7.9 ng $ml^{-1}$) than the benign cell line (1.3 ng $ml^{-1}$). CAM assay detected a VEGF-dependent angiogenic activity in the medium from malignant cells, but only a relatively weak VEGF-independent activity in the medium from benign cells. These results demonstrated that malignant cells did over-express VEGF and only the VEGF derived from malignant cells was angiogenically active. Thus, we suggest that the VEGF produced by malignant cells might play an important role in facilitating metastasis of prostatic cancer. © 2000 Cancer Research Campaign

**Keywords:** vascular endothelial growth factor; prostatic cell lines; over-expression; metastasis; isoforms

Adenocarcinoma of the prostate is now the second leading cause of male death from malignant disease in the US and in Europe (Foster, 1990; Geller, 1995). In common with other malignant diseases, metastasized prostate cancer cells are invariably resistant to all currently available therapies (Foster et al, 1992). This glaring clinical fact has lent fresh impetus to the need for better understanding of mechanisms underlying metastatic behaviour. Progress on this front has been heightened recently by reproducible models of inducing metastases through transfection of fragmented DNA from malignant tumours into non-metastatic cells (Ke et al, 1998). Identification and characterization of genes involved in the metastatic phenotype of prostate cancer cells through these model systems hold the promise of propelling much needed advancements in therapy.

Whilst the metastatic cascade may involve complicated multiple genetic changes, angiogenesis is an essential common mechanism for the development and the formation of metastasis in solid tumours. This makes anti-angiogenic therapy an attractive and promising treatment for cancer (Battegay, 1995). Angiogenesis is regulated by a number of both stimulating and inhibiting angiogenic factors. VEGF is one of the most potent factors for stimulating angiogenesis (Stephan and Brock 1996). Although VEGF has been previously detected in prostate cancer (Jackson et al, 1997; Ferrer et al, 1998; Haggstrom et al, 1998), these observations were made in tissue specimens. Thus, it is not clear whether the detected VEGF is made by tumour cells themselves or by their adjacent cells. This is important because VEGF has been reported to be produced by tumour-associated cells like endothelial cells, infiltrated T-cells and macrophages, as well as by surrounding stromal cells (Fukumura et al, 1998). Furthermore, it is not completely understood whether it is the VEGF or other angiogenic factors, such as basic fibroblast growth factor (FGF) and interleukin-8, which have also been detected in some prostate tissues (Deshmukh et al, 1997; Haggstrom et al, 1998), that are responsible for neovascularization required for the malignant progression of prostate cancer cells. There is no experimental data to show whether the VEGF produced by a human prostate carcinoma cell line (Ferrer et al, 1997) is capable to induce angiogenesis.

In order to make a quantitative assessment of differential expression of VEGF between the benign and the malignant prostate epithelial cells, and particularly, to test the angiogenic ability of the VEGF produced by prostate carcinoma cells, we have performed the present study in well-characterized cell culture system. We have first measured the level of VEGF expression in a wide range of different human and rat prostate cell lines with either benign or malignant properties to determine whether the elevated expression of VEGF is associated with the increased malignant characteristics of the cells. Then, we have examined

*Received 27 September 1999*
*Revised 19 January 2000*
*Accepted 25 January 2000*

*Correspondence to:* HJ Chen whether the ability of producing high level of VEGF can be transferred as the transformation of the metastatic capability of the cells by comparing the VEGF levels in three metastatic variants with that in their parental benign cells. These metastatic variants have been generated by transfection of a benign cell line with genomic DNA extracted from prostate carcinoma cells (Ke et al, 1998). Finally, we employed an in vivo angiogenesis assay to determine whether the VEGF produced by the malignant cells can induce new blood vessel formation.

### MATERIALS AND METHODS

### Cell lines and culture conditions

Three groups of 13 well-characterized benign and malignant cell lines were used in this study. The first group is the four human prostate cell lines which include one benign cell line and three malignant cell lines. The benign cell line PNT-2 was derived from human prostate epithelial cells (Cussenot et al, 1991, 1994; Berthon et al, 1995). One of the malignant cell line LNCap was derived from prostate carcinoma (Horoszewicz et al, 1983), and other two malignant cell lines, PC-3 and DU145, were derived from malignant metastases of prostate cancer in rib and brain respectively (Kaighn et al, 1978; Stone et al, 1978). The second group of cell lines examined in this study was the five biologically distinct sublines from the Dunning R3327 rat prostate carcinoma model (Isaacs et al, 1986). The G cell line is the least malignant cell line and it can induce only less than 5% of animals with metastasis when introduced into the syngeneic rats. The second cell line in this group was the weakly metastatic cell line AT-2, which can induce up to 20% of animals with metastasis. The remaining three cell lines in this group were the highly metastatic cell lines AT-3, MAT-Lu and AT-6.1, which can induce more than 75% of animals with metastasis. The last group of cell lines used in this study was the benign rat cell line Rama 37 (Dunnington et al, 1983) and its three malignant metastatic sublines established by transfecting the Rama 37 cells with DNA extracted from prostate carcinoma cells (Ke et al, 1998). The human and rat cell lines were grown as monolayer cultures in RPMI-1640 (human cells) and Dulbecco's modified Eagles medium (DMEM) (rat cells) media, supplemented with 10% (v/v) fetal calf serum, hydrocortisone (5 ng $ml^{-1}$), penicillin (100 units $ml^{-1}$) and streptomycin (100 mg $ml^{-1}$), with either testosterone (5 ng $ml^{-1}$, for human and rat prostate cells), or insulin (5 ng $ml^{-1}$, for Rama 37 and its derivative lines).

### cDNA probes

The human VEGF probe used in this study was a 655-bp cDNA fragment (a gift from ZENECA Pharmaceuticals). Both glyceraldehyde 3-phosphate dehydrogenase (GAPDH) and actin probes were purchased from CLONTECH Laboratory (UK). The rat VEGF probe was a 601-bp cDNA fragment prepared by reverse transcription polymerase chain reaction (RT-PCR), using 100 ng mRNA extracted from AT-3 cells as template, and using a pair of rat VEGF specific primers (sense: 5′-AACCATGAACTTTCT-GCTCTC-3′; antisense: 5′-GGTGAGAGGTCTAGTTCCCGA-3′). The sequence of the amplified rat VEGF probe was confirmed by nucleotide sequencing analysis using a DNA sequencing kit (Amersham Life Science). Each probe containing 25 ng cDNA was labelled with [$\alpha$-$^{32}$P]dCTP to a specific radioactivity of $0.5–1.0 \times 10^9$ dpm $\mu g^{-1}$ cDNA using a random promed DNA labelling kit (Boehringer Mannheim GmbH, Germany).

### Detection of VEGF mRNA

The expression of VEGF mRNA in different cell lines were detected by Northern blotting. Total RNA was isolated from each cell line by lysing the cells with 4 M guanidine isothiocyanate and purified by CsCl gradient centrifugation. The mRNA was further purified from total cellular RNA using Oligotex mRNA Kit (Qiagen Ltd, UK). Northern blotting was performed as described previously (Alwine et al, 1977). Briefly, total RNA (10 μg) was separated by electrophoresis in a 0.8% denaturing formaldehyde-agarose gels. The separated RNAs were transferred onto a nylon Hybond-N membrane (Amersham Inc, UK) overnight by capillary elution. After the membranes were pre-hybridized at 42°C for 3–4 h in the solution containing 50% formamide, 5% Denhardt's, 6 × sodium–saline phosphate–EDTA (SSPE), 0.5% sodium dodecyl sulphate (SDS) and 100 μg $ml^{-1}$ of denatured sonicated salmon sperm DNA, the membranes were hybridized overnight under the same condition with cDNA probes. The probed membranes were subjected to stringent washes and exposed to Kodak XAR-5 films. The intensity of each band appearing on the film was scanned and analysed with Phoretix 1D-advanced vs 3.1 software. The loading artifact was normalized with the constitutively expressed GAPDH or actin mRNAs.

Slot blotting was used for quantitative analysis of VEGF mRNA expressed in four human prostate cell lines. Increasing amounts of mRNA from 0.05 to 1.6 μg were loaded onto a nylon Hybond-N membrane using a slot-blot apparatus (Bio-Rad, Hercule, CA, USA). After fixed by ultraviolet light, the membrane was pre- and hybridized with the radioactively labelled human VEGF probe and GAPDH probe respectively, washed and subjected to autoradiography. Intensities of each band were scanned as described above for Northern blotting. The best-fit straight lines of plots of peak area against the amount of RNA per slot over the linear part of the plot were calculated by linear regression. Any loading artefacts were normalized with the consititutively expressed GAPDH mRNA.

### Detection of VEGF protein

In a 96-well microtitre plate, 100 μl of mouse anti-hVEGF monoclonal antibody (1 μg $ml^{-1}$ in 50 mM of sodium carbonate, pH 9.6) was coated overnight at 4°C. The plate was then blocked with blocking buffer (2% bovine serum albumin (BSA) in phosphate-buttered saline (PBS)) for 2 h at room temperature (RT). Conditioned medium (serum-free medium used for culturing cells for 48 h) or rhVEGF standard (R & D System) was added and incubation continued for another 2 h at RT. Goat anti-hVEGF polyclonal antibody (R & D System) was added (100 μl of 1 μg $ml^{-1}$) to each well after washing the plate with the washing buffer (0.1% Tween-20 in PBS) and then incubated at RT for 2 h. The detection antibody, a peroxidase-labelled rabbit anti-goat IgG (Zymed, USA, 61-1620), was added and reaction continued for another 2 h at RT. The plate was washed again with washing buffer, the substrate solution (0.04% o-phenylenediamine dihydrochloride in PBS plus 0.4 ml $l^{-1}$ of 30% hydrogen peroxide) was used to develop colour. The colour was quantitated with TiterteK Multiskan PLUS (Flow Laboratories, Finland) at 492 nm. The limit of sensitivity of this assay was 0.4 ng $ml^{-1}$.

### Analysis of isoforms of VEGF gene transcripts

RT-PCR analysis was performed to study potential splicing variants of the VEGF gene in human cell lines. The first strand of

© 2000 Cancer Research Campaign cDNA was synthesized from the poly-adenosine (poly-A) end of the mRNA (100 ng) extracted from both PNT-2 and DU145 cells using the First Strand cDNA Synthesis Kit for RT-PCR (Boehringer). The reverse transcription products (2.5 µl) were amplified by PCR using a primer pair allowing to amplify all possible VEGF isoforms (sense: 5′-TCGGGCCTCCGAAAC-CATGC-3′; antisense: 5′-CCTGGTGAGAGATCTGGTTC-3′). The amplified PCR products were subjected to electrophoresis and visualized by ethidium bromide staining. The products were also transformed onto a nylon membrane (Hybond N, Amersham) and hybridized with the human VEGF probe. To confirm the sequences of products amplified by PCR being VEGF isoforms, the PCR products were cloned into pBlueScript plasmid, and their sequences were determined by nucleotide sequencing analysis using a DNA Sequencing Kit (Amersham Life Science).

### Chick chorioallantoic membrane assay

The angiogenic activity of the conditioned media from both the malignant DU145 and the benign PNT-2 cells were determined by the CAM assay, as described previously (West et al, 1985). Both control medium (serum-free medium without cells) and conditioned medium were concentrated tenfold by centrifugal ultra-filtration (3 kDa cut-off). The rhVEGF 121 protein (positive control) was dissolved in Dulbecco's PBS at a concentration of 100 ng ml$^{-1}$. For application to the eggs, a 40 µl aliquot of each sample was mixed with 50 µl of 1% sterile methylcellulose (M-0512, 4000 centipoises, Sigma) and 1 µl PBS or 1 µl anti-hVEGF neutralizing antibody (MAB293, R & D Systems Ltd) at a 50-fold excess concentration to rhVEGF 121. This mixture (10 µl) was then applied on to a 2 mm diameter 'Teflon' column and dried, under sterile conditions, to give a clear disc. The samples were applied on to the CAM on day 10, when vessel growth ostensibly finished. The angiogenic reaction was determined on day 14. The angiogenic reaction was scored as: 0 – negative; 0.5 – change in vessel architecture but not directed to the point of sample application; 1 – partial spokewheel (1/3 of circumference exhibits directional angiogenesis); 2 – spokewheel, 3 or up–strong and fully spokewheel. This approach enabled calculation of accumulated responses for the test samples in each group. For photography, the membranes were fixed in situ with ice-cold 4% paraformaldehyde-PBS that was injected both from above and below the membrane. Membranes were excised, placed on a fresh microscope slide and photographed under a Leitz binocular dissecting microscope and indirect fibreoptic illumination. Statistical analysis was performed using the Mann–Whitney *U*-test.

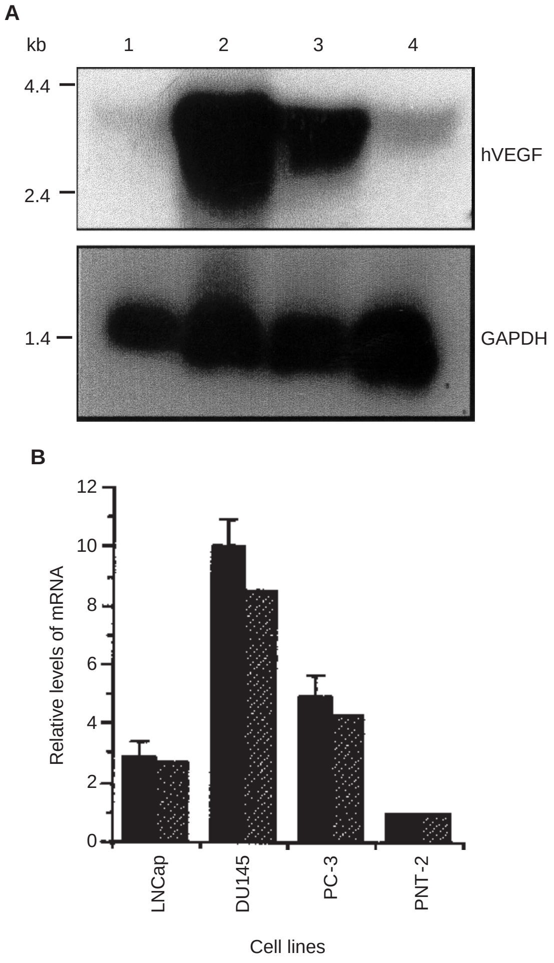

**Figure 1** Detection of VEGF transcripts in benign and malignant human prostate cell lines. (**A**) Northern blotting. Total RNA (about 10 µg) from LNCap (1), DU145 (2), PC-3 (3) and PNT-2 (4) were subjected to electrophoresis in a 0.8% agarose gel. The separated RNAs were transferred onto a nylon membrane and hybridized with radioactively labelled human VEGF cDNA probe. The washed membrane was exposed to a Kodak XAR-5 film for 3 days. The same membrane was re-hybridized with a radioactively labelled GAPDH cDNA probe and the hybridized band visualized by exposure to Kodak film for 3 h. Molecular size of RNA markers are shown in kilobases (kb). (**B**) Quantification of the relative levels of VEGF mRNA. The relative hybridization of the VEGF transcripts to the VEGF cDNA probe was determined by measuring the intensity of autoradiographic images of the Northern and Slot-blots. Black bars represent the mean and standard deviation (s.d.) of three measurements by three separate slot-blot analyses. The shaded bars represent single measurement by Northern blotting

### RESULTS

### VEGF mRNA level in the benign and malignant cell lines

Northern and slot blotting was used to detect the level of VEGF mRNA in human benign and malignant cell lines. The radioactively labelled human VEGF probe detected one VEGF band at a molecular size of about 3.9 kilobases (kb) in all the four cell lines examined (Figure 1A), consistent with the expected size of VEGF mRNA (Tischer et al, 1991; Shima et al, 1995). Scanning the intensities of the bands showed that the lowest level of VEGF mRNA was detected in the benign cell line PNT-2, this level was increased by 2.7-fold in the malignant cell line LNCap. Further increases of 4.3-fold and 8.5-fold were detected in the malignant metastasis-derived cell lines PC-3 and DU145 respectively. More accurate measurements of the VEGF mRNA level by slot-blotting detected similar differences between the benign and the malignant cell lines as those detected by Northern blotting. Relative to the lowest level expressed in the PNT-2 cell line, the level of VEGF mRNA in LNCap, PC-3 and DU145 cell lines was increased by $2.9 \pm 0.5$, $4.9 \pm 0.7$ and $10 \pm 0.95$-fold respectively (Figure 1B).

The levels of VEGF mRNA in the five Dunning R3327 rat carcinoma cell lines were measured using Northern blotting. As shown in Figure 2A, the expression of VEGF in these cell lines was elevated in direct correlation with the increase in their metastatic characteristics. The G-cell line, which has the lowest metastatic potential, expressed the lowest level of VEGF mRNA.

© 2000 Cancer Research Campaign

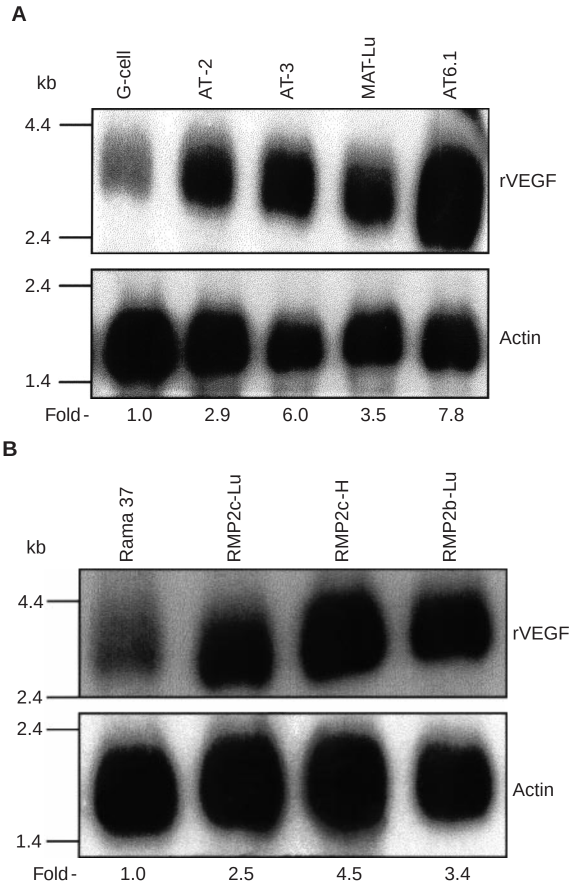

**Figure 2** Detection of VEGF transcripts in the Dunning R3327 rat cell lines (**A**), and Rama 37 and its derivative cell lines (**B**) by Northern blotting. Total RNA (about 10 μg) from each cell line was subjected to electrophoresis in a 0.8% agarose gel. The separated RNAs were transferred onto a nylon membrane and hybridized with radioactively labelled rat VEGF cDNA probe. The washed membrane was exposed to a Kodak XAR-5 film for 5 days. The same membrane was re-hybridized with a radioactively labelled rat actin cDNA probe and visualized by exposure to Kodak film overnight. The relative hybridization was determined by measuring the intensities of the bands with densitometry scanning

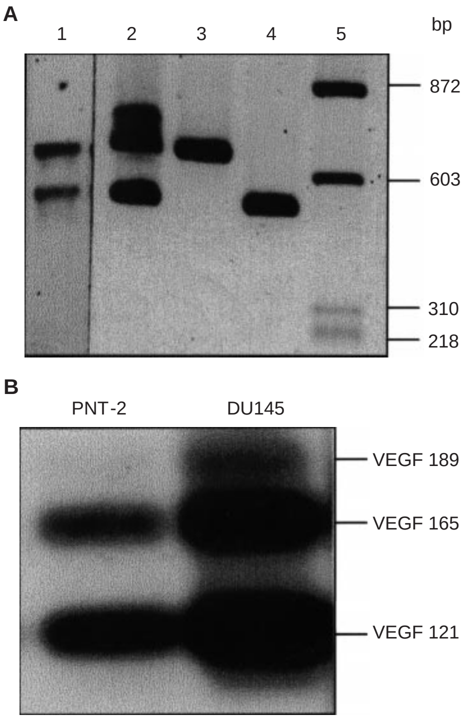

**Figure 3** Detection of the isoforms of VEGF in benign and malignant human cell lines. (**A**) Detection by RT-PCR. The first-strand cDNA was synthesized from 100 ng mRNA extracted from PNT-2 (1) and DU145 cells (2) respectively. The amplified PCR products were electrophoresed in a 0.8% agarose gel. VEGF 165 (3) and VEGF 121 (4) DNA fragments were included as controls. Lane 5 is the DNA size markers. (**B**) Southern-blotting analysis of the VEGF isoforms. The RT-PCR products amplified from the cDNA of PNT-2 and DU145 cells were electrophoresed in an agarose gel, transferred onto a nylon membrane and hybridized with radioreactively labelled human VEGF cDNA probe. The radioactivity was visualized by radioautography

The VEGF expression level was 2.9-fold higher in the weakly metastatic AT-2 cells than that in the G-cells. In the three highly metastatic cell lines, AT-3, MAT-Lu and AT-6.1, the expression of VEGF mRNA was 6-, 3.5- and 7.8-fold respectively higher than that expressed in the G-cells.

The levels of VEGF mRNA in the benign rat Rama 37 cell line and its three malignant metastatic sublines were shown in Figure 2B. The lowest level was detected in the benign Rama 37 cells. Comparing with their parental benign Rama 37 cells, the amount of VEGF mRNA expressed in the RMP2c-Lu, RMP2c-H and RMP2b-Lu was increased by 2.5-, 4.5- and 3.4-fold respectively.

### Detection of the VEGF isoforms in human benign and malignant cell lines

RT-PCR performed in the benign PNT-2 and the malignant DU145 cells (Figure 3A) showed that two VEGF mRNA isoforms coding for VEGF 121 and VEGF 165 proteins, respectively, were transcribed in both the benign (lane 1) and the malignant (lane 2) cell lines. In addition to these two smaller transcripts, another band with a molecular size larger than the VEGF 165 transcript was detected in the malignant DU145 cells, but not in the benign PNT-2 cells (Figure 3B). Nucleotide sequence analysis revealed that the smallest band was VEGF 121, the middle-sized band was VEGF-165 and the largest band was VEGF 189.

### VEGF protein secreted by human benign and malignant cell lines

At the protein level (Figure 4), the difference in VEGF synthesis between the benign and the malignant cell lines was observed. The VEGF protein secreted by the benign PNT-2 cells ($8 \times 10^5$ cells) was 1.3 ± 0.06 ng per 48 h. In contrast, the amounts of VEGF protein secreted in malignant LNCap cells and the malignant metastasis-derived PC-3 cells and DU145 cells were 2.7 ± 0.03 ng, 3.1 ± 0.09 ng and 7.9 ± 0.09 ng respectively, under the same culture condition.

### Angiogenic activity of the VEGF protein produced by human benign and malignant cell lines

Conditioned media from a benign (PNT-2) and a malignant cell line (DU 145) cell line were collected for CAM assay, using rhVEGF 121 as the positive control. As shown in Figure 5A and 5D, the rhVEGF 121 induced a strong angiogenic response which was neutralized by a 50-fold excess of VEGF neutralizing antibody. The

*© 2000 Cancer Research Campaign* 

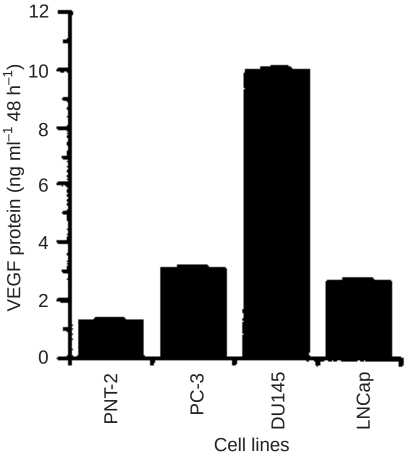

**Figure 4** Detection of VEGF proteins secreted by human benign and malignant cell lines with ELISA. The level of VEGF in conditioned media from the benign and the malignant cell lines were measured using ELISA. The amount of VEGF protein was calculated by relating to the standard curve established from serial dilutions of rhVEGF protein. Bars represent the mean and standard deviation (s.d.) of six measurements from two separate experiments response to conditioned medium from PNT-2 cells was weak (Figure 5C), but conditioned medium from DU145 cells showed a moderate angiogenic response (Figure 5B). The VEGF antibody completely neutralized the angiogenic effect of the conditioned medium from malignant DU145 cells (Figure 5E). However, there was no effect of the same antibody on the weakly angiogenesis induced by the conditioned medium from benign PNT-2 cells (Figure 5F). Quantitative analysis of angiogenesis induced by the above different additions was performed using the criteria described in Materials and Methods and shown in Figure 5G.

### DISCUSSION

In this study, we have investigated the expression of VEGF in several benign and malignant cell lines from both human and rat cell models. Among the four human prostate cell lines, the weakly metastatic cell line LNCap expressed nearly three times the level of VEGF mRNA and twice the level of VEGF protein as that expressed in the benign PNT-2 cells. In the two malignant metastasis-derived cell lines, PC-3 and DU145, the expression of VEGF mRNA was fourfold and tenfold respectively higher than that expressed by the benign PNT-2 cells. These results suggest that the enhanced expression of the VEGF gene is associated with the malignant characteristics of human prostate cells, which is consistent with previous published works (Ferrer et al, 1997; Connolly and Rose 1998; Balbay et al, 1999; Melnyk et al, 1999).

A similar pattern of VEGF expression was also detected in the rat cell lines used. In the weakly metastatic cell line AT-2, the expression level of VEGF was nearly three times of that detected in the least malignant G-cells; whereas the expression of VEGF in the three highly metastatic cell lines AT-3, MAT-Lu and AT6.1 was 3.5- to 7.8-fold higher than that detected in the G-cells. These results not only show that the high level expression of VEGF was associated with the malignant phenotype of the prostatic epithelial cells, but that the elevated expression of VEGF was closely associated with the increasing metastatic characteristics of the malignant cells. As such, it is possible that VEGF may play an important role in the malignant dissemination of prostate cancer cells.

Higher levels of VEGF in serum or plasma have been evaluated as a predictor of outcome in patients with several malignant diseases (Dirix et al, 1996; Crew et al, 1997; Salven et al, 1997; Kraft et al, 1999; Molica et al, 1999). Recently, it has also been suggested that the patients with metastatic prostate cancer have higher plasma VEGF levels than those with localized disease or healthy controls (Duque et al, 1999). Over-expression of VEGF by malignant prostate carcinoma cells as shown by our investigation and other reports (Ferrer et al, 1997; Connolly and Rose 1998; Balbay et al, 1999; Melnyk et al, 1999) may contribute to the elevated serum or plasma VEGF levels. More studies are needed to confirm the predictive utility of the VEGF produced by malignant prostate cells themselves.

In this study, we also examined the isoform patterns of VEGF mRNA expressed by the cells with either benign or malignant characteristics. Five different isoforms of VEGF transcripts encoding polypeptides of 206, 189, 165, 145 and 121 amino acids have been reported to be expressed in human cells (Houck et al, 1991; Tischer et al, 1991; Poltorak et al, 1997). Our results revealed that VEGF 121 and 165 were the predominant VEGF isoforms expressed by both benign and malignant human cell lines. Interestingly, an additional transcript representing VEGF 189 isoform was only detected in the malignant cells, but not in the benign cells. Recently, it was reported that colon cancer patients with liver metastasis showed significantly higher levels of VEGF 189 mRNA than those without liver metastasis. The expression of VEGF 189 isoform may be correlated with metastasis and a poorer prognosis in colon cancer (Tokunaga et al, 1998). Combining this finding with our observation, the absence of VEGF isoform 189 in the benign PNT-2 cells may indicate that the increasing malignant characteristics of prostate carcinoma cells may not only be associated with the elevated level of VEGF, but also correlated with the expressed isoform patterns of VEGF gene. Further studies are needed to establish whether the expression of a particular VEGF isoform (e.g. VEGF 189) in malignant cells could be used clinically to predict the stage of prostate carcinoma or the outcome of patients with the disease.

Results from CAM assay demonstrated that the process of new blood vessel formation was markedly stimulated by conditioned medium from malignant DU145 cells, and this activity was completely abolished by a neutralizing anti-hVEGF antibody. Conversely, the weaker angiogenic activity induced by conditioned medium from the benign PNT-2 cells was not inhibited by the addition of the same neutralizing antibody. These results indicated that the moderate angiogenic activity of malignant cells was caused by the highly expressed VEGF protein whereas the low angiogenic activity observed in the conditioned medium from benign cells was caused by factors other than VEGF. This suggests that not only the level of VEGF was greatly increased in the malignant cells, but also the VEGF produced by the malignant cells played a greater, or pivotal role in stimulating angiogenesis than that produced by the benign cells. Thus, the elevated amount of angiogenically active VEGF derived from malignant cells may be responsible for the development of neovascularization in the microenvironment of secondary tumours. Since angiogenic activity can facilitate the process of cell metastasis (Liotta et al, 1991; Weidner, 1995), it is possible that the metastatic capability of the malignant prostate cells may have been greatly facilitated by the high expression of biologically active VEGF, with its strong angiogenesis-promoting activity.

© 2000 Cancer Research Campaign

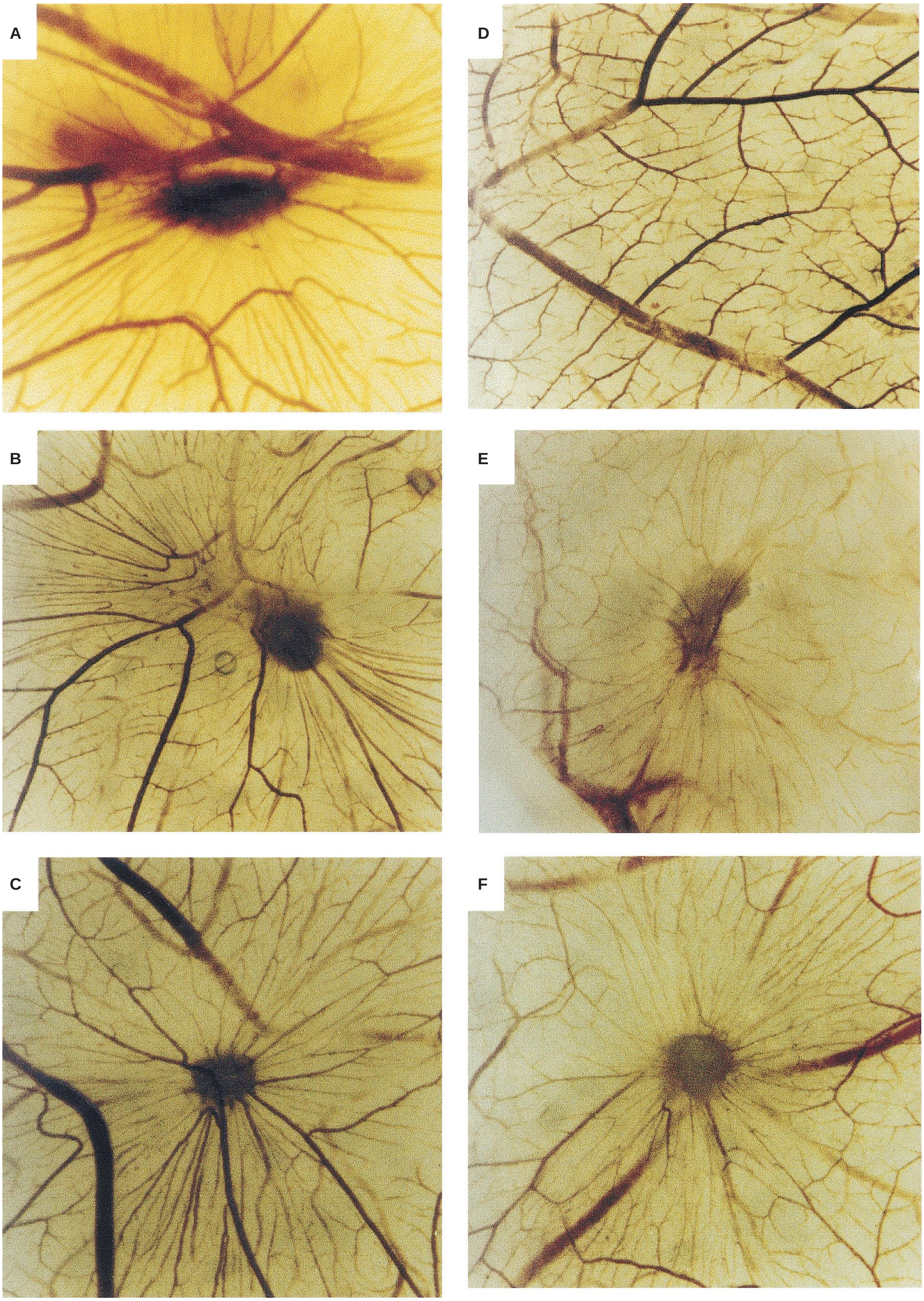

**Figure 5** Detection of angiogenic activity by CAM assay. Angiogenesis in chick embryos was induced by rhVEGF 121 protein (**A**), the conditioned media from the malignant metastasis-derived cell line DU145, (**B**) and the benign cell line PNT-2 (**C**). The inhibition of the angiogenic activity induced by rhVEGF 121 (**D**), and conditioned medium from the malignant DU145 cells (**E**) was achieved by the VEGF neutralizing antibody. However, the same antibody did not affect the weak angiogenic activity induced by the conditioned medium from the benign PNT-2 cells (**F**). All pictures are ×8 magnification. (**G**) a plot of CAM score of the angiogenic activities in the presence of the different additions, and '+' indicates the medium plus the neutralizing anti-hVEGF antibody. *Represents $P < 0.05$ vs control and **represents $P < 0.05$ vs sample without the neutralizing antibody (Mann–Witney *U*-test)

*© 2000 Cancer Research Campaign*

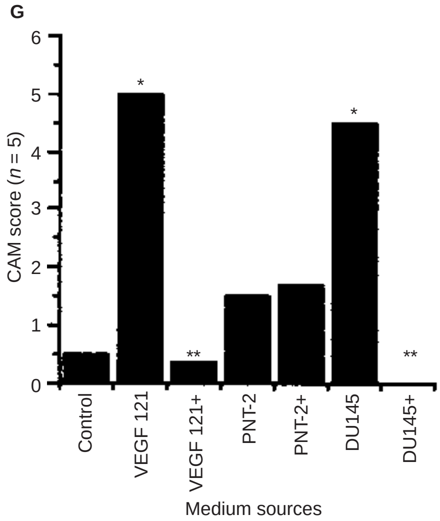

**Figure 5** continued

To further test this hypothesis, we compared the expression levels of VEGF mRNA between the benign Rama 37 cell line (Dunnington et al, 1983) and its three metastatic variants, RMP2b-Lu, RMP2c-Lu and RMP2c-H. Northern blotting showed that the level of VEGF mRNA expressed in three metastatic cell lines were 2.6- to 4.8-fold higher than that expressed by their benign parental Rama 37 cells. This result indicated that both the capability of synthesizing high level VEGF and of disseminating to secondary sites, which are possessed by the DNA donor cells AT6.1, had been transformed into the DNA recipient cells Rama 37 through DNA transfection. Further studies determining how the metastasis-promoting DNA from the donor cells up-regulates VEGF expression in the recipient cells could provide much needed insight into the molecular mechanism for the initiation and development of malignant progression in prostate and other malignant diseases.

Although we have generally found that the elevated expression of VEGF was closely associated with the increased metastatic potential of the cells, we observed a possible discrepancy between the malignant PC-3 and DU145 cell lines. It has been demonstrated that the metastatic potential of PC-3 is slightly higher than that of DU145 cells (Ke et al, unpublished observation). However, in this work, the VEGF expression in DU145 was higher than that in PC-3, although both were much higher (ten- and nearly fourfold respectively) than that expressed by the benign PNT-2 cells. Since PC-3 was established from a rib metastasis (Kaighn et al, 1979) and DU145 was established from a brain metastasis (Stone et al, 1978), the different growing microenvironments of the secondary tumours may have different requirements on the speed and degree of vascularization. More importantly, metastasis is the consequence of complicated molecular and genetic changes occurring in malignant cells. Whilst elevated expression of VEGF may be important in promoting and maintaining metastasis development, there may be more gene(s) involved in the initiation of malignant dissemination. Our recent work (accepted for publication by *Cancer Research*.) suggested that it is likely that the collective effect of several metastasis-related genes, rather than VEGF alone, determined the behaviour of the individual malignant cells. Although the over-expression of VEGF is important and seems to be essential for metastasis, the metastatic capability of the cells is not completely determined by VEGF alone. Thus, the discrepancy in VEGF expression observed between PC-3 and DU145 cells may reflect the differences in molecular complexities which caused the spread of the two separate clones to different secondary sites in the body.

In conclusion, we have now provided evidence to support a role for the angiogenically active VEGF derived from the malignant cells as a metastasis-associated gene in prostate cancer. Its relevance in the relative comparison between prostate cell lines of differing metastatic potential was further emphasized in the rat metastatic model of prostate cancer. VEGF ligand secreted by malignant prostate cells may be a key factor in inducing angiogenesis through paracrine mechanisms on the surface of endothelial cells and in so doing, greatly facilitate the process and development of metastasis.

### ACKNOWLEDGEMENTS

The authors are grateful to Dr Roy Bicknell and ZENECA Pharmaceuticals for providing the VEGF 121 and VEGF 165 cDNAs, Peter Baker for the scanning analysis and Tracy Awbery for preparation of the manuscript.

### REFERENCES

Alwine JC, Kemp DJ and Stark G (1977) Methods for the detection of specific RNAs in agarose gel by transfer to diazobenzymethyl-paper and hybridization with DNA probes. *Proc Natl Acad Sci USA* **12**: 5350–5354

Balbay MD, Pettaway CA, Kuniyasu H, Inoue K, Ramirez E, Li E, Fidler IJ and Dinney CPN (1999) Highly metastatic human prostate cancer growing within the prostate of athymic mice overexpression vascular endothelial growth factor. *Clin Cancer Res* **5**: 783–789

Battegay EJ (1995) Angiogenesis-mechanistic insights, neovascular diseases, and therapeutic prospects. *J Mol Med* **73**: 333–346

Berthon P, Cussenot O, Hopwood I, Leduc A and Maitland NJ (1995) Functional expression of SV40 in normal human prostatic epithelial and fibroblastic cells – differentiation pattern of non-tumorigenic cell-lines. *Int J Oncol* **6**: 333–343

Connolly JM and Rose DP (1998) Angiogenesis in two human prostate cancer cell lines with differing metastatic potential when growing as solid tumors in nude mice. *J Urol* **160**: 932–936

Crew JP, O'Brien T, Bradburn M, Fuggle S, Bicknell R, Cranston D and Harris AL (1997) Vascular endothelial growth factor is a predictor of relapse and stage progression in superficial bladder cancer. *Cancer Res* **57**: 5281–5285

Cussenot O, Berthon P, Berger R, Mowszowixz I, Faille A, Hojman F, Teillac P, Leduc A and Calvo F (1991) Immortalization of human adult normal prostatic epithelial-cells by Liposomes containing Large T-SV40 gene. *J Urol* **146**: 881–886

Cussenot O, Berthon P, Cochandpriollet S, Maitland NJ and Leduc A (1994) Immunocytochemical comparison of cultured normal epithelial prostatic cells with prostatic tissue-sections. *Exp Cell Res* **214**: 83–92

Deshmukh N, Scotson J, Dodson AR, Ke Y and Foster CS (1997) Differential expression of acidic- and basic-FGF in benign prostatic hyperplasia identified by immunohistochemistry. *Br J Urol* **80**: 869–874

Dirix LY, Vermeulen PB, Hubens G, Benoy I, Martin M, De Pooter C and Van Oosterom AT (1996) Serum basic fibroblast growth factor and vascular endothelial growth factor and tumour growth kinetics in advanced colorectal cancer. *Ann Oncol* **7**: 843–848

Dunnington DJ, Kim U, Hughes CM, Monaghan P, Ormerod J and Rudland PS (1983) Phenotypic instability of rat mammary tumor epithelial cells. *J Natl Cancer Inst* **71**: 1227–1240

Duque JLF, Loughlin KR, Adam RM, Kantoff PW, Zurakowski D and Freeman MR (1999) Plasma levels of vascular endothelial growth factor are increased in patients with metastatic prostate cancer. *Urol* **54**: 523–527

Ferrer FA, Miller LJ, Andrawis RI, Kurtzman SH, Albertson PC, Laudone VP and Kreutzer DL (1997) Vascular endothelial growth factor (VEGF) expression in human prostate cancer: in situ and in vitro expression of VEGF by human prostate cancer cells. *J Urol* **157**: 2329–2333

*© 2000 Cancer Research Campaign*

Ferrer FA, Miller LJ, Andrawis RI, Kurtzman SH, Albertson PC, Laudone VP and Kreutzer DL (1998) Angiogenesis and prostate cancer: in vivo and in vitro expression of angiogenesis factors by protate cancer cells. *Urol* **51**: 161–167

Foster CS (1990) Predictive factors in prostatic hyperplasia and neoplasia. *Hum Pathol* **21**: 575–577

Foster CS and Abel PD (1992) Clinical and molecular techniques for diagnosis and monitoring of prostatic cancer. *Hum Pathol* **23**: 395–401

Fukumura D, Xavier R, Sugiura T, Chen Y, Park EC, Lu N, Selig M, Nielsen G, Taksir T, Jain R and Seed B (1998) Tumor induction of VEGF promoter activity in stromal cells. *Cell* **94**: 715–725

Geller J (1995) Approach to chemoprevention of prostate-cancer. *J Clin Endocrinol Met* **80**: 717–719

Haggstrom S, Wikstrom P, Bergh A and Damber JE (1998) Expression of vascular endothelial growth factor and its receptors in the rat ventral prostate and Dunning R3327 PAP adenocarcinoma before and after castration. *Prostate* **36**: 71–79

Horoszewicz JS, Leong SS, Kawinski E, Karr JP, Rosenthal H, Chu TM, Miranol EA and Murphy GP (1983) LNcap model of human prostatic-carcinoma. *Cancer Res* **43**: 1809–1818

Houck KA, Ferrara N, Winer J, Cachianes G, Li B and Leung DW (1991) The vascular endothelial growth factor family: identification of a fourth molecular species and characterization of alternative splicing of RNA. *Mol Endocrinol* **5**: 1806–1814

Isaacs JT, Issaacs WB, Feitz WFJ and Scheres J (1986) Establishment and characterization of seven Dunning rat prostatic cancer cell-lines and their use in developing methods for predicting metastatic abilities of prostatic cancer. *Prostate* **9**: 261–281

Jackson MW, Bentel JM and Tilley WD (1997) Vascular endothelial growth factor (VEGF) expression in prostate cancer and benign prostatic hyperplasia. *J Urol* **157**: 2323–2328

Kaighn ME, Narayan S, Ohnuki Y, Lechner JF and Jones LW (1979) Establishment and characterization of a human prostatic carcinoma cell line (PC-3). *Invest Urol* **17**: 16–23

Ke Y, Beesley C, Smith, Barraclough R, Rudland PS and Foster CS (1998) Generation of metastatic variants by transfection of a rat non-metastatic epithelial cell line with genomic DNA from rat prostatic carcinoma cells. *Br J Cancer* **77**: 287–296

Kraft A, Weindel K, Ochs A, Marth C, Zmiji J, Schumacher P, Unger C, Marme D and Gastl G (1999) Vascular endothelial growth factor in the sera and effusions of patients with malignant and nonmalignant diseases. *Cancer* **85**: 178–187

Liotta LA, Steeg PS, Stetler-Stevenson WG (1991) Cancer metastasis and angiogenesis: an inbalance of positive and negative regulation. *Cell* **64**: 327–336

Melnyk O, Zimmerman M, Kim KJ and Shuman M (1999) Neutralizing anti-vascular endothelial growth factor antibody inhibits further growth of established prostate cancer and metastasis in a pre-clinical model. *J Urol* **161**: 960–963

Molica S, Vitelli G, Levato D, Gandolfo GM and Liso V (1999) Increased serum levels of vascular endothelial growth factor predict risk of progression in early B-cell chronic lymphocytic leukaemia. *Br J Haematol* **107**: 605–610

Poltorak Z, Cohen T, Sivan R, Kandelis Y, Spira G, Vlodavaky I, Keshet E and Neufeld G (1997) $VEGF_{145}$, a secreted vascular endothelial growth factor isoform that binds to extracellular matrix. *J Biol Chem* **272**: 7151–7158

Salven P, Maenpaa H, Orpana A, Alitalo K and Joensuu H (1997) Serum VEGF is often elevated in disseminated cancer. *Clin Cancer Res* **6**: 647–651

Shima DT, Adamis AP, Ferrara N, Yeo KT, Allende R, Folkman J and Damore PA (1995) Hypoxic induction of endothelial-cell growth-factors in retinal cells – identification and characterization of vascular endothelial growth-factor (VEGF) as the mitogen. *Mol Med* **2**: 64–71

Stephan CC and Brock TA (1996) Vascular endothelial growth factor, a multifunctional polypeptide. *Puerto Rico Health Sci J* **15**: 169–178

Stone KR, Mickey DD, Wunderli H, Mickey GH and Paulson DF (1978) Isolation of a human prostate carcinoma cell line (DU-145). *Int J Cancer* **21**: 274–281

Tischer E, Mitcher R, Hartman T, Silva M, Gospodarowicz D, Fiddes JC and Abraham JA (1991) The human gene for vascular endothelial growth factor, multiple protein forms are encoded through alternative exon splicing. *J Biol Chem* **266**: 11947–11954

Tokunaga T, Oshika Y, Abe Y, Ozeki Y, Sadahiro S, Kijima H, Tsuchida T, Yamazaki H, Ueyama Y, Tamaoki N and Nakamura M (1998) Vascular endothelial growth factor (VEGF) mRNA isoform expression pattern is associated with liver metastasis and poor prognosis in colon cancer. *Br J Cancer* **77**: 998–1002

Weidner N (1995) Intratumor microvessel density as prognostic factor in cancer. *Am J Pathol* **147**: 9–15

West DC, Hampson IN, Arnold F and Kumar S (1985) Angiogenesis induced by degradation products of hyaluronic-acid. *Science* **228**: 1324–1326

*© 2000 Cancer Research Campaign*
